# Does Playing Tennis with a Low-Compression Ball Effect Psychophysiological Responses and Match Characteristics in Recreational Adult Players?

**DOI:** 10.3390/sports12030080

**Published:** 2024-03-13

**Authors:** Bulent Kilit, Ersan Arslan, Yusuf Soylu, Andrew M. Lane

**Affiliations:** 1 Faculty of Sport Sciences, Tokat Gaziosmanpasa University, Tokat 60250, Turkey; bulent.kilit@gop.edu.tr (B.K.); ersan.arslan@gop.edu.tr (E.A.); yusuf.soylu@gop.edu.tr (Y.S.); 2 Psychological Research Centre, School of Psychology, University of Wolverhampton, Wolverhampton WV1 1LY, UK

**Keywords:** tennis, modified equipment, slower balls, notational analysis, lc-ball induced

## Abstract

This study aimed to compare the effects of playing tennis using low-compression balls (Lc-Balls) and standard balls (St-Balls) on psychophysiological responses and match characteristics among recreational adult tennis players. Participants (N = 24; age: 20.5 ± 1.3 years) were randomly matched to play two singles matches over three sets: one match was played with a Lc-Ball and one match was played with a St-Ball, resulting in twenty-four matches. Heart-rate responses and match characteristics were assessed during each match. Post-match measures included retrospective assessments of perceived exertion, ratings of enjoyment towards physical activity, and ratings of mental effort and mood. Results showed higher psychophysiological responses and more intensive play during the game when playing with the Lc-Ball (*p* ≤ 0.05, d values ranging from 0.24 to 1.93 [small to very large effect]). Further, playing with a Lc-Ball related to reporting a lower rating of perceived exertion (*p* = 0.00, d = 0.90 [moderate effect]) and greater physical enjoyment (*p* = 0.00, d = 1.73 [large effect]). Playing with the St-Ball was associated with higher unpleasant mood responses including depression, tension, anger, and fatigue. In conclusion, the results suggest that using the Lc-Ball may lead to better match performance with higher enjoyment in the tennis match-play in recreational adult tennis players.

## 1. Introduction

Tennis is a racket sport that involves anaerobic activities such as high-intensity sprints, dynamic turns, and jumps. Players perform short, high-intensity workouts over a long period, interspersed with rest or low-intensity activities [1,2,3,4]. Various factors such as different court surfaces (clay, hard, and grass) with a variety of ball types (fast, medium, and slow) and skill levels have been shown to effect both the physiological responses and performances of tennis players [1,5,6,7,8].

Research examining the effects of playing simulated tennis matches has shown that technical and match characteristics are influenced by using different pieces of equipment, including low-compression balls (Lc-Balls), shorter racquets, and changing the size of the court [9]. Ball compression is color coded. A green ball is 25% slower than a yellow ball; an orange ball is 50% slower than a yellow ball, and a red ball is 75% slower than a yellow ball. Significantly longer rallies are associated with playing with the Lc-Ball when compared to playing with standard balls (St-Balls) [10]. As a result of this, rally length in children increased, who were also novices, when using a Lc-Ball when compared to a St-Ball [10,11]. Among children, the smallest-scale equipment combination (a small racket with a red ball) produced significantly better hitting performances than other racket-and-ball combinations [12,13]. Furthermore, the Lc-Ball was found to have the most significant positive effect on simulated tennis match performance. Using a Lc-Ball during the simulated tennis match provides a significant longer rally with less movement between strokes. Therefore, this tool allows players to stroke the ball with more force. Consequently, this situation supports the development of improved tennis skills during challenging learning environments such as training and simulated tennis matches. Considering these results, the equipment that should be used to teach tennis to children, and thus by extension, novice players across all ages groups, should be reconsidered [10,11,12,13].

In recent years, a number of different approaches have been used to help skill development and performance including Touch Tennis, Tennis Xpress, and Play and Stay [14,15]. The primary purpose of these alternative approaches to playing the game is to increase the length of rallies. Longer rallies provide feedback. Such technical changes will have the greatest effect among players in the relatively early stages of learning to play the sport. Although, numerous studies examined technical responses and match characteristics across various court surfaces, scaled equipment, and performance levels [9,10,11,12]. However, no studies have investigated the effects of different ball compression on these important performance variables of recreational adult players. To our knowledge, the present study is the first to examine these variables in detail in recreational adult tennis players. We hypothesized that recreational players would demonstrate more enjoyment levels with better hitting performances using low-compression balls when compared to playing with standard balls.

The physical and mental health benefits of physical activity are well known, with the challenge being to raise physical activity levels. Recent research has demonstrated the benefits of experiencing pleasant emotional responses to increasing physical activity [16,17,18]. Further, when people exercise at a high intensity, as evidenced by reporting a high rating of perceived exertions, then physiological sensations dominate consciousness. This sense of intense physiological effort is associated with unpleasant emotions. Although there is evidence that some athletes prefer intense exercise, if increasing physical activity among a largely sedentary population is a goal, then increasing enjoyment by considering reducing the intensity of a session is desirable [16,17,18]. When applied to tennis balls, it is suggested that this is associated with playing longer rallies, as there is more time to prepare to hit the ball, and as such, this will be associated with greater enjoyment, lower ratings of perceived exertion, and improved mood. Therefore, the aim of the present study was to compare the effect of playing tennis using different ball compressions on psychophysiological responses and match characteristics in recreational adult players.

## 2. Materials and Methods

### 2.1. Participants

Sample size was estimated using the G*Power software (G-Power, version 3.1.9.7, University of Dusseldorf, Dusseldorf, Germany). After adding a partial effect size of 0.30, a power of 0.8, and a *p*-value of 0.05 (two groups and two measurements) for a correlation of 0.5, a recommended total sample size of 24 was required. Therefore, twenty-four recreational adult male volunteer tennis players were recruited (Table 1). To control for the possible effects of left-handed players’ performance, all players were right-handed. All matches involved playing against another right-handed player. All participants were familiar with the training workload of 2–3 training sessions per week (120–180 min.week^−1^). The inclusion criteria were (i) an age of 18–34 years and (ii) to have been playing tennis regularly, involving playing competitive match performances (singles or doubles) for a minimum time of six months. Participants were excluded if there were (i) acute injuries or (ii) non-attendance at tennis matches. After informing participants of research procedure and potential risks, written informed consent was provided. This study was conducted following the principles of conducting ethical research, as outlined in the Declaration of Helsinki [19] and the protocol approved by the Ethics Committee (1 J .01.2023/02.06) of the university of the first author.

### 2.2. Experimental Design

A randomized cross-over design was used to compare the effects of Lc-Ball and St-Ball on psychophysiological responses and match characteristics in recreational adult tennis players. The study was performed over two main experimental sessions: (1) on-court assessments, including (HTTT) and (ITN), and (2) simulated two-singles tennis-match playing. To avoid fitness level and technical skill mismatches during match-play, all pairs were balanced according to their VO_2max_ and technical scores. Players performed tennis matches in two singles, for the best of three sets: randomly, one game with the Lc-Ball “green balls” (green 25% slower than the yellow ball) and one match with the St-Ball “yellow balls” [20].

Every session started with a standardized 10 min warm-up section consisting of running and tennis-specific actions. During each match, data were captured that assessed heart rate (HR), percentage of heart rate (%HR), and match characteristics—strokes per rally (SPR), duration of the rally (DR), rest time between in-point (RT), effective playing time (EPT), average match time (AMT), average stroke per match (ASPM), and average game per match (AGPM). These data were continuously monitored and recorded during twenty-four tennis matches. Furthermore, data were assessed that recorded a rating of perceived exertion (RPE), physical activity enjoyment scale (PACES), a rating scale of mental effort (RSME), and the Profile of Mood States (POMS). These data were assessed at the end of each match.

All tests and matches were performed on a standard indoor hard-court surface. All tennis matches took place at a similar time (between 09:00 a.m. and 11:00 a.m.) day, and similar temperatures (20–23 °C) and relative air humidity levels (30–45%) were obtained through all stages of the study. To minimize any adverse effects of physical and physiological fatigue on singles tennis-match performances, each match was played with at least 72 h between each match [21]. The players were told to maintain regular nutritional intake before and during the simulated tennis matches.

### 2.3. The Hit-and-Turn Tennis Test

The test, an acoustically controlled progressive (hitting a sequence of forehand and backhand strokes) on-court fitness test for tennis players, was carried out following the techniques proposed in this study [22]. HR_max_ was the maximum HR measurement observed during the test. Following the HTTT, the estimated VO_2max_ for adult men was calculated using the following formula: VO_2max_ = 30.0 + 2.00 × (player finishes the level in the HTTT).

### 2.4. International Tennis-Number Test

The International Tennis Federation (ITF) uses the ITN ranking system to measure the performance of tennis players at all levels. This system, which includes serve, forehand–backhand groundstrokes, forehand–backhand volley depth, accuracy, and mobility evaluation, is a commonly used test in the tennis-specific literature to measure on-court game characteristics [23]. All participants viewed the ITN test protocols video before the test, and the best test result was recorded after two attempts [24]. A ball machine (Tennis Tutor Plus, Burbank, CA, USA^®^) was employed to feed balls to the tested players.

### 2.5. Tennis Match Analysis and Psychophysiological Measures

All participants were randomly matched to play singles tennis matches after a standard 10 min warm-up program (running and tennis-specific actions such as forehand and backhand, volleys, and serves). All twenty-four matches (n = 12, Lc-Ball; n = 12, St-Ball) were played into the rules of the ITF (the best of 3 sets) [25]. A set of 3 new yellow balls and green balls was used for each match. All matches were played on a standard indoor hard-court surface (GreenSet) on full-size standard tennis courts. The participants used their own tennis racquets and were accustomed to playing competitive matches with both balls [20]. A portable HR-monitoring device (Polar V800 Polar OY, Kempele, Finland^®^) was used to record each player’s HR responses during the matches. Each player’s match was recorded using two video cameras (Sony HDR—CX240 Full HD, Tokyo, Japan^®^) positioned 2 m from the side of the court at the service-line level and approximately 6 m above the court for the duration of the match [3].

A specialized movement-specific analysis program, Kinovea (version 0.8.15; Kinovea, Bordeaux, France), was used to analyze the matches, and the analysis of all of the matches was performed by the same experienced researcher [26]. The observer was tested for reliability using a test–retest protocol of analyzing and coding the parameters twice, interspaced by 20 days. The intra-class correlation test (ICC) revealed a value of 0.96, suggesting an excellent reliability level. Based on match data, the following variables were calculated for each game: DR, RT (not including changes of ends), EPT (expressed as a percentage of the total time when the ball was in play during a game), SPR, AMT, ASPM, and AGPM [26]. RPE responses (6–20) were also assessed at the end of each match [27].

After each match, the short form of PACES was used to measure the enjoyment levels of players [28]. This scale, which includes five items scored on a 1–7 Likert scale, has been validated as an indicator of enjoyment level in training in Turkish players [29]. The internal consistency of the modified 5-item scale was found to be 0.78. The rating scale for mental effort (RSME) was used to assess the subjective mental effort during each match. The scale is reported to have good validity and face validity [30]. It is a single item, and participants find it easy to use. It assesses mental workload, and scores range from 0 (no effort) to 150 (extreme effort) [31]. The POMS [32] is a commonly used model of mood. The original POMS comprises 65 items and assesses six dimensions of mood: tension, anger, confusion, depression, fatigue, and vigor. The POMS was completed before and after each tennis match. A Turkish version of POMS was used to evaluate mood profiles when asking, “How do you feel right now?” [33] and therefore captures mood in the context of playing the tennis match rather than other response timeframes such as ‘past week’, which can assess emotions experienced over a range of different situations. POMS scale was in the range of 0.81–0.91.

### 2.6. Statistical Analysis

The data are reported as means and standard deviations. Before parametric tests, normality assumption was confirmed using the Shapiro–Wilk test (n < 30). A paired t-test was performed with a Bonferroni correction on each dependent variable and was used to compare psychophysiological responses and match characteristics between Lc-Ball vs. St-Ball during simulated tennis-match play. Partial eta squared ($\eta_{p}^{2}$) and Cohen’s *d* were calculated for each dependent variable. $\eta_{p}^{2}$ was considered small (0.01–0.06), moderate (0.06–0.14) or large (>0.14), and Cohen’s d was considered trivial (<0.2), small (0.2–0.6), moderate (0.6–1.2), large (1.2–2.0), very large (2.0–4.0), and nearly perfect (>4.0) [34]. Also, the 95% Confidence Interval (95% CI) was calculated for the difference between mean values for each of the estimated variables. A repeated measure analysis of variance was used to compare mood states before and after the Lc-Ball vs. St-Ball. Inter-individual variability in psychophysiological responses and match characteristics between the Lc-Ball vs. St-Ball matches was quantified using the coefficient of variation (CV%). The intra-class correlation coefficient (ICC) was used to determine the test–retest reliability of the match characteristics. Statistical analyses were performed with IBM SPSS Statistics for Windows software version 21.0 (IBM Corp.^®^, Armonk, NY, USA, released 2012). The significance level was determined as *p* < 0.05.

## 3. Results

Playing tennis with a Lc-Ball was associated with higher psychophysiological responses in terms of HR (t = 10.292; *p* = 0.000; 95% CI: −16.15 to −10.90), %HR_max_ (t = 22.322; *p* = 0.000; 95% CI: −16.05 to −13.45), and PACES (t = 3.794; *p* = 0.000; 95% CI: −8.03 to −2.13), except for RPE (t = 6.626; *p* = 0.000; 95% CI: 1.38 to 2.57) and RSME (t = 3.227; *p* = 0.004; 95% CI: 16.75 to −3.66) in Table 2.

In contrast to RT (t = 2.047; *p* = 0.042; 95% CI: 0.19 to 10.97) and AGMP (t = 2.399; *p* = 0.035; 95% CI: −4.15 to 0.17), the Lc-Ball resulted in higher match characteristics in Table 3.

The mood responses of the recreational adult tennis players to playing Lc-Ball vs. St-Ball matches are contained in Table 4. The results demonstrated that using the St-Ball induced higher unpleasant mood responses in terms of higher scores of depression, tension, anger, and fatigue (*p* ≤ 0.05, $\eta_{p}^{2}$ = ranging from 0.081 to 0.314 (trivial to small effect)).

## 4. Discussion

The present study compared the effects of Lc-Balls and St-Balls on psychophysiological responses and match characteristics among recreational adult tennis players. The results revealed that playing tennis with the Lc-Ball was associated with higher psychophysiological responses including a higher HR, %HR, and PACES when compared to playing with the St-Ball. Playing with the Lc-Ball was associated with playing longer rallies, a pleasant mood, a lower rating of perceived exertion, and greater enjoyment. Playing tennis with the Lc-Ball led to differences in the way matches were played, with a change in the rhythm of the game, and was ultimately noticed by spending more time on strokes (the flying duration of the ball). Therefore, these factors might help players be able to perform more controlled and accurate strokes and have better control of their ball-hitting technique, the speed, and the direction. Arguably, these factors are highly beneficial to players learning to play with the ball.

A growing body of evidence demonstrates that playing variables such as scaled equipment, gender, skill levels, and court surfaces (grass, hard, and clay) influence tennis performance [5,6,7,35]. Furthermore, evidence shows that all these variables have also been shown to effect tennis players’ technical actions [12,35]. It is not easy to compare our results against previous studies due to the absence of similar studies. We argue that there is a need for more research, as the present line of enquiry suggests that there are beneficial effects for recreational tennis players. Of available studies that could be used for a comparative purpose, we use evidence from a younger group of tennis players. For example, on slower surfaces (clay court), playing time, total match time, and average rally time is longer, resulting in increased physical strain from a large number of strokes and short- and high-intensity activities [5,7,35]. In addition, clay court surfaces, including longer rallies and shorter rest times, led to performing a slower game and shot rhythm and increased HR and higher blood–lactate levels and higher PACES responses in comparison to playing on a hard court surface [5,36]. The different results from the court-surface characteristics might explain the game rhythm during match-play. Contrary to higher RPE responses, the players on clay court surfaces had more time to stroke the ball or prepare to play than on hard court surfaces [6,35]. Our results showed that playing with the St-Ball induced more perceived physical strain, resulting in increased mental effort and negative mood responses such as depression, tension, anger, and fatigue. These findings can be related to the adverse effect of game performances and increased mental effort during match play.

Our findings show that playing tennis with modified equipment (a Lc-Ball, shorter racquets, and smaller court dimensions) effects tennis-match performance responses such as technical skills and match-play characteristics [9,10]. For instance, a study found that children playing with St-Balls and field sizes made fewer strokes than mini-tennis-match playing [10]. Furthermore, with the increase in the DR and SPR of children in the game, the technical actions cause a more balanced and correct style to be learned [11]. Additionally, scaling equipment provides better performance in match-play conditions, as well as optimizing the working environment [20,37,38,39,40,41]. The results in the literature showed that the playing style during the match with the Lc-Balls (the red, orange, and green balls) was more dynamic, aggressive, and faster than (longer rallies and fewer errors) with the St-Ball [38,39,40]. Moreover, the tennis matches made with mini-tennis stages and rules for children showed that the red and orange stages scored the most in the average number of rallies [38,40]. The main reason is that the distance to be moved with the Lc-Ball is shorter because of the longer rally times and the smaller playing field during the game [38]. In addition, it was seen that modified ball handling increased rally speed and average strokes per rally, as well as players hitting at a lower height (slower bounce) [20,38,41]. This game rhythm provides the players with more time to hit so that more strokes can be performed in rallies [20,40]. The Lc-Ball allowed players to rally at speeds that more closely resembled those expected of adult tennis matches [20,41]. Increasing the number of hits during training might be related to more challenging learning environments and superior tennis-skill development in young tennis players [10,41]. In addition to these results, it was determined that playing with modified equipment provided more enjoyment than playing with standard materials for children [10,41]. Recent studies have been performed to confirm the execution of modification equipment used for enhancing children’s technical proficiency and the ability to maintain a rally [38,39].

We argue that these findings are consistent with previous research on children [10,11,38,39]. As a result of these studies, scaled equipment might improve technical skill, enjoyment, and involvement in tennis, especially in young tennis players [10,11]. The last study indicated that using scaled equipment positively affects the development of technical skills in adult beginner tennis players [42]. However, adults’ skill levels and physical abilities may differ according to children’s. Despite the knowledge gained in the literature, the biggest challenge is determining how and when to move from scaled equipment to standard equipment for adults and children [42,43]. So, further research is needed to investigate the effectiveness of scaled equipment in skill development or match performance for adults. Given this sample size, care should be taken in generalizing the results to all adult tennis players. As a limitation of this study, only 24 young adult male recreational players were included in our study. Therefore, our results cannot generalize to female recreational tennis players. In addition, different performance responses can be obtained using modified equipment for athletes of different ages, genders, and skill levels. Moreover, we conducted this study on hard courts, and performance assessments can be made on tennis courts with different surfaces. Finally, more research is needed on recreational adult tennis players to determine whether modified equipment provides better playing experiences.

We argue that a strength of the present study is in the rigorous methods used, which enhance internal validity but have an ecologically valid context. First, internal validity was strengthened as we standardized the playing ability. This meant that one player was not clearly better than the other, and so fewer points would be won by serving an ace or returning a winner when returning a serve. Second, we measured multiple performance factors, with each adding unique information on how the nature of the match changed. Third, we assessed physiological and psychological responses to playing and so argue that this approach when seen collectively was rigorous. In terms of ecological validity, players competed in real games where the intention was to win, and therefore, players would intend to a win a game via serving an ace or playing a winning passing shot. We encourage future research to consider the relative internal and ecological validity in their designs, as this approach could lead to evidence that could translate research to practice.

Findings from the present study have implications for players learning tennis due to findings which point to a way of enhancing enjoyment and improving mood. Recreational tennis players could be motivated for physical and health-related reasons. There is a wealth of evidence to support the benefits of exercise on physical and mental health. If someone chooses tennis as the activity to improve their physical health, then clearly playing longer rallies will be more beneficial than one-shot points. It is feasible that for novice players, the challenge is to hit the ball in the court, and be able to return the ball. With multiple factors effecting performance, the present study showed that providing more time to hit the ball via changing the ball can have a meaningful influence. The present study finds evidence to support the fact that mood and enjoyment are enhanced. Future research should look at the longitudinal effects and whether the choice of ball predicts adherence.

The findings of the present study have some practical implications for tennis coaches and sports scientists. Using a Lc-Ball in simulated tennis matches may be a valuable strategy to enhance recreational adult tennis players’ psychophysiological responses, match characteristics, and increase their enjoyment and motivation levels. Practitioners and coaches may consider using Lc-Balls as an alternative or supplementary tool to improve recreational adult tennis players’ technical and tactical skills, especially beginners and intermediates. Based on this information and the literature, using Lc-Balls for children and recreational adult players can make their tennis game experience easy and much more fun.

## 5. Conclusions

Although the use of modified equipment for children has been recommended recently, it needs to be clarified which modifications are optimal for recreational adult tennis players of different ages and skill levels. This is the first study to examine tennis matches played with St-Balls and Lc-Balls in recreational adult tennis players. Our findings suggest that playing tennis with a Lc-Ball causes stronger psychophysiological responses such as in HR and PACES with a lower RPE. In addition, recreational adult tennis players playing with Lc-Balls can positively be affected by their match performances. With these results, we recommend using Lc-Balls in matches for recreational adult tennis players, like with children.

## Figures and Tables

**Table 1:** Subjects’ characteristics.

Variable	Age (Years)	Body Height (cm)	Body Mass (kg)	VO_2max_ (mL.kg^−1^.min^−1^)	ITN Scores	Tennis Experience (Years)
Mean (SD)	20.5 (1.3)	174 (3.4)	65.4 (4.4)	55.3 (1.3)	8 (1)	1.2 (0.4)

**Table 2:** The psychophysiological responses of participants.

	Lc-Ball M SD	CV (%)	St-Ball M SD	CV (%)	Mean Difference	Cohens *d* and Rating
HR (beat.min^−1^)	157 ± 16 *	10.2	143.5 ± 17	11.8	13.5	0.80; Moderate
%HR (%)	76 ± 7.6 *	10.0	61.5 ± 7.4	12.0	14.5	1.93; Large
RPE	13.2 ± 2.2	16.7	15.2 ± 2.2 Ω	14.5	−2.0	0.90; Moderate
PACES	30.2 ± 3.0 *	9.9	25.1 ± 2.9	11.5	5.1	1.73; Large
RSME	92.7 ± 19.3	20.8	102.9 ± 18.5 Ω	18.0	−10.2	0.54; Small

HR = heart rate; %HR = percentage of average heart rate; RPE = rating of perceived exertion; PACES = physical activity enjoyment scale; RSME = rating scale of mental effort. * Significant difference from St-Ball; Ω significant difference from Lc-Ball.

**Table 3:** The match characteristics of participants.

	Lc-Ball M SD	CV (%)	St-Ball M SD	CV (%)	Mean Difference	*95% CI*	Cohens *d* and Rating
SPR (n)	6.1 ± 4.1 *	67.2	3.5 ± 2.8	80.0	2.6	−3.4/−1.7	0.73; Moderate
DR (s)	11.9 ± 5.8 *	48.7	6.5 ± 5.3	81.5	5.4	−6.6/−4.2	0.97; Moderate
RT (s)	24.2 ± 22.8	94.2	29.8 ± 23.7	79.5	−5.6	0.2/10.9	0.24; Small
EPT (%)	26.2 ± 2.8 *	10.7	22.3 ± 3.7	16.6	3.9	−5.6/−2.1	1.16; Moderate
AMT (min)	67.1 ± 12.4 *	18.5	56.7 ± 11.2	19.7	10.4	−19.1/−1.7	0.88; Moderate
ASPM (n)	556.7 ± 93.3 *	16.8	404.6 ± 87.9	21.7	152.1	−212.9/−91.2	1.67; Large
AGPM (n)	19.1 ± 3.4	17.8	16.9 ± 2.8	16.6	2.2	−4.1/−0.2	0.70; Moderate

SPR = strokes per rally; DR = rally duration; RT = rest time; EPT = effective playing time; AMT = average match time; ASPM = average stroke per match; AGPM = average game per match; * Significant difference from St-Ball.

**Table 4:** The mood responses of participants.

	Tennis Matches			Time			Interaction		
	F_(1,23)_	P	$\eta_{p}^{2}$	F_(1,23)_	*p*	$\eta_{p}^{2}$	F_(1,23)_	*p*	$\eta_{p}^{2}$
Depression	7.376	0.009 *	0.138	5.073	0.029 *	0.099	4.036	0.050 *	0.081
Tension	0.892	0.350	0.019	8.671	0.005 *	0.159	18.716	0.000 *	0.289
Anger	20.396	0.000 *	0.307	18.172	0.000 *	0.283	21.102	0.000 *	0.314
Vigor	0.690	0.410	0.015	4.315	0.043	0.086	0.936	0.338	0.020
Fatigue	315.415	0.000 *	0.873	22.669	0.000	0.330	11.402	0.002 *	0.199
Confusion	1.887	0.176	0.039	0.059	0.810	0.001	0.432	0.514	0.009
TMD	24.306	0.000 *	0.346	3.368	0.043 *	0.068	9.532	0.003	0.172

TMD: Total mood disturbance; * significant difference from Lc-Ball. *p* < 0.05.

## Data Availability

The data are available from the corresponding author upon reasonable request.
